# The Editor's Letter-Box

**Published:** 1915-03-06

**Authors:** A. H. Coughtrey


### THE EDITOR'S LETTER-BOX.

### The Education of Hospital Officers.

*To the Editor of* The Hospital.

Sir,—You say in your Editorial of February 6 that the hospital committees "who prefer non-hospital men to junior officers when filling the higher positions, must have found disadvantages outweighing the value of previous experience in the candidates whom they passed over." You then point out, I think somewhat irrelevantly and unconvincingly, that it is difficult to maintain a high status among junior officers if you admit such to a professional society or organisation except as a privilege for having achieved a kind of rank in their profession.

Now, Sir, really is it in the least likely that these committees have any such reason for passing over candidates with hospital experience? For instance, in the appointment under discussion two of the selected candidates, as well as many of the non-selected applicants, were men of tried experience, and, in fact, had held important administrative appointments in hospitals, and this surely removes any doubt as to their efficiency.

They were not rejected because they belonged to this or that society or organisation—it is doubtful if the committee gave a thought to this matter even if it had the slightest idea that they were members of a society; and, in any case, this could not have been the reason, or they would not have been among the selected candidates at all—but I think we may take it that they were rejected for reasons quite apart from their efficiency or suitability for the post, which was unquestionable.

In order to take an impartial view of the matter, it should be recognised that these appointments are given by the rule of majority. The parties may be so evenly divided that the absence of even one gentleman may convert what may have been a majority into a minority. It is quite possible, again, that a candidate may be personally known to the majority of a committee, or to one who can influence a majority, who may be anxious to secure his election, but in this case it is certainly unfortunate if this majority does not show its hand until after an advertisement, which may disqualify its man, has been issued.

But, after all, appointments such as these are quite infrequent, and give little cause for dismay to the junior officer, whose principal aim should be to gather experience in administration which would make unquestionable his eligibility for a superior post. "There is nothing in war," said Napoleon, "which I cannot do by my own hands. If there is nobody to make gunpowder, I can manufacture it. The gun-carriages I know how to construct. If it is necessary to make cannons at the forge, I can make them. The details of working them in battle, if it is necessary to teach, I shall teach them. In administration it is I alone who have arranged the finances." In like manner efficiency should be the watchword of the junior who aspires to the chief administrative post in a hospital, and to gain this efficiency he should make himself acquainted with the conditions of every detail of hospital work.

The educational scheme of the Incorporated Association of Hospital Officers has been designed for this purpose, and the juniors who avail themselves of it will have in their hands a lever which may well turn a committee in their favour when seeking higher appointments. Much advantage in their hospital education may likewise be derived from their membership of a professional association.

But it must not be forgotten that the committee of election has the final word, and unless the committees can be persuaded to give us their ideas as to what constitutes the eligible man, and themselves adhere to their own conditions, we shall undoubtedly hear now and again that men with no hospital experience are placed in important posts at these institutions.—Yours faithfully,

February 24, 1915. A. H. Coughtrey.

[We did not suggest that hospital candidates were passed over because they belonged to this or that professional society, but that such societies were not providing the best training which admitted all and sundry to membership. That such societies largely exist to improve the training of junior hospital officials the educational scheme alluded to is sufficient to show.—Ed. The Hospital.]

